# Utilisation of eight or more antenatal care visits and its associated socio-economic-related inequalities in sub-Saharan Africa: A decomposition analysis

**DOI:** 10.1371/journal.pone.0312412

**Published:** 2025-03-25

**Authors:** Richard Gyan Aboagye, Augustus Osborne, Anayochukwu Edward Anyasodor, Sharon Vera Yikindi, Qorinah Estiningtyas Sakilah Adnani, Bright Opoku Ahinkorah

**Affiliations:** 1 School of Population Health, University of New South Wales, Sydney, New South Wales, Australia; 2 Department of Family and Community Health, Fred N. Binka School of Public Health, University of Health and Allied Sciences, Hohoe, Ghana; 3 Department of Biological Sciences, School of Basic Sciences, Njala University, PMB, Freetown, Sierra Leone; 4 Rural Health Research Institute, Charles Sturt University, Orange, New South Wales, Australia; 5 Cape Coast Nursing and Midwifery Training College, Cape Coast, Ghana; 6 Department of Public Health, Faculty of Medicine, Universitas Padjadjaran, Bandung, West Java, Indonesia; 7 REMS Consultancy Services Limited, Sekondi-Takoradi, Western Region, Ghana; 8 Faculty of Health and Medical Sciences, The University of Adelaide, Adelaide, Australia; VART Consulting PVT LTD, INDIA

## Abstract

**Introduction:**

Inadequate utilisation of maternal healthcare services, particularly antenatal care (ANC), poses a challenge in sub-Saharan Africa (SSA). There is a dearth of regional studies that address the socio-economic disparities in the use of ANC in SSA. Therefore, we examined the wealth and education-based inequalities in the utilisation of ANC services among women in SSA.

**Methods:**

We analysed secondary data obtained from the Demographic Health Survey conducted in fifteen countries in SSA. We estimated the degree of wealth and education-related inequalities using concentration curves, concentration indices (CIX), and decomposition analysis, which identified the factors contributing to the disparities in the utilisation of ANC. All the analyses were conducted using Stata version 17.0 (Stata Corporation, College Station, TX, USA).

**Results:**

The results revealed a significant socio-economic gap in utilising ANC in SSA. We found positive and statistically significant wealth index-related (CIX = 0.30; p-value < 0.0001) and education-based inequalities (CIX = 0.33; p-value < 0.0001) in eight or more ANC visits. The extent of wealth index-related and education-based inequalities varied across the fifteen countries. The decomposition analysis showed that educational attainment accounted for about 21% of the inequalities in eight or more ANC visits. Wealth index contributed 12.14% of the inequalities in eight or more ANC visits. Our results further showed that women's education, wealth, parity, and place of residence significantly contributed to the utilisation of eight visits or more among women in SSA.

**Conclusion:**

This study shows the disparities in ANC coverage, contingent upon wealth index and educational attainment. Our study highlights the importance of adopting a holistic approach involving robust cooperation between healthcare and other social service sectors. It is crucial to prioritise the primary social factors contributing to disparities in the utilisation of ANC services, including women’s education, parity, place of residence, and economic status. Policymakers and stakeholders must prioritise efforts to combat obstacles to healthcare access, including the provision of easily accessible, affordable, and culturally appropriate services.

## Introduction

Maternal mortality rates in several countries globally still pose a challenge [1], despite global efforts to significantly reduce maternal deaths by 2030 [2], highlighted in target 3.1 of the Sustainable Development Goals [3]. About 287,000 global maternal deaths occurred in 2020, which translates to approximately 800 maternal deaths daily, implying that one woman died every two minutes from preventable pregnancy-related causes [2,4]. The burden of adverse maternal health outcomes continues to be significant in various low-and middle-income countries [5], with countries in sub-Saharan Africa (SSA) contributing 70% of global maternal deaths [2]. Despite the decrease in the maternal mortality ratio (MMR) to 34.2% between 2000 and 2020, MMR remains a significant concern in the African region, including SSA [6]. The majority of maternal mortality stems from avoidable or treatable pregnancy or childbirth-related complications or manageable conditions [7, 8]. It has been reported that the significant complications responsible for 75% of all maternal fatalities include pregnancy-related infection, severe obstetric haemorrhage, hypertensive disorders during pregnancy (pre-eclampsia and eclampsia), delivery complications, unsafe abortion, and prolonged or obstructed labour [6,9]. However, these issues do not often receive the desired attention due to disparities in antenatal care (ANC) utilisation [10–12]. These inequalities threaten the new guidelines on ANC, which aim to ensure a positive pregnancy experience [13, 14], and leaves poorer women in SSA behind [12,15], further widening inequality gaps in ANC use [16].

In SSA, at least one sub-national region lacks a minimum of one hospital for every half a million population [17], indicating the remarkable healthcare inequalities in the region. The gaps in accessing maternal healthcare services are a challenge of an unequal world [18]. Therefore, understanding the drivers of disparities in ANC services utilisation in SSA becomes critical in achieving a substantial decrease in maternal mortality by 2030 [2]. Several factors have been associated with maternal ANC services utilisation and categorised into community-level characteristics, socio-demographic and pregnancy-related attributes, media exposure, and the presence of maternal health services [19]. The utilisation of ANC is primarily influenced by maternal education, marital status, women’s employment, husband’s education, accessibility, household income, cost, birth interval, previous obstetric complications experienced by pregnant women, and timely visits [20, 21].

In addressing socio-economic inequalities in access to quality maternal healthcare services utilisation, ANC is essential in attaining Universal Health Coverage [22]. Extensive studies have highlighted the disproportionate utilisation of maternal healthcare services among women of diverse socio-economic levels and rural-urban dwellings [23–26]. There are indications that women from higher socio-economic classes are increasingly inclined to utilise healthcare institutions [27–29]. In Africa, one of the significant barriers preventing women from using maternal health services is geographic accessibility [30]. Information on socio-economic disparities in ANC is critical to establishing interventional strategies, advocating equity in maternal health, and advancing maternal and child health outcomes [12]. The preceding signals the World Health Organization (WHO) and major stakeholders to concentrate efforts towards achieving the 2030 MMR reduction target [6]. Appreciating the relevance of healthcare services rendered to pregnant women and how these services are accessed and utilised based on socio-economic status will, in no small measure, enhance efforts in tackling maternal deaths in SSA. These services include medical check-ups, screenings, and educational sessions, among other interventions [31–33]. The WHO recommends a minimum of eight ANC contacts (ANC visits) for optimal outcomes in perinatal mortality and improved women’s care experience [13]. The importance of eight or more ANC visits and better quality ANC visits extends to the facilitation of uptake of preventive strategies, prompt detection of risk factors, and reduction of complications, while addressing health disparities [13].

To the best of our knowledge, no study has holistically addressed the socio-economic inequalities in ANC services utilisation in SSA. This study is set to make such a contribution by decomposing the socio-economic inequalities in ANC services utilisation among women in SSA using the standard decomposition technique. The findings of our study will be crucial for policy-making and maternal healthcare priority setting in SSA and other parts of the world.

## Methods

### Data source

We utilised data from fifteen (15) countries in SSA. The data was extracted from the recent Demographic and Health Survey (DHS) conducted in each of the 15 countries spanning from 2018 to 2022. These countries had data on all the variables of interest as at the time of the study. The detailed methodology regarding the DHS, including the sampling technique, data collection instrument, and procedure can be found in the literature [34, 35]. Briefly, the DHS is a nationwide survey conducted in several low- and middle-income countries to advance an understanding of health and population trends [34]. The DHS adopted a cross-sectional design, and the respondents were sampled using a multistage sampling technique. A structured and pretested questionnaire was used to collect data from the respondents: men, women, and children on several health and sociodemographic issues, including ANC services utilisation [35]. Our study included a sample of 112,123 women of reproductive age (15–49 years) across 15 countries in SSA. Table 1 contains a list of the countries included in the study with their corresponding survey years and weighted samples. Only women with a birth history before the DHS and those with observations on all variables of interest were included in the study. We followed the Strengthening the Reporting of Observational Studies in Epidemiology (STROBE) guidelines in writing this paper (S1 Table) [36].

**Table 1:** Description of the study sample.

Countries	Year of survey	Pooled weight (N)	Weighted percentage
1.Burkina Faso	2021	8,374	7.5
2.Benin	2017–18	7,662	6.8
3.Cote d’Ivoire	2021	6,621	5.9
4.Cameroon	2018	7,195	6.4
5.Gabon	2019–21	5,160	4.6
6.Gambia	2019–20	5,266	4.7
7.Guinea	2018	5,170	4.6
8.Liberia	2019–20	3,646	3.2
9.Madagascar	2021	8,960	8.0
10.Mali	2018	5,241	4.7
11.Mauritania	2019–21	7,593	6.8
12.Nigeria	2018	20,146	18.0
13.Rwanda	2019–20	7,165	6.4
**14.**Sierra Leone	2019	7,410	6.6
**15.**Zambia	2018	6,514	5.8
**All countries**	**2017–2022**	**112,123**	**100.0**

### Variables

The number of ANC visits was the outcome variable in this study. To assess this variable, women who had given birth in the last 5 years were asked to indicate the number of ANC sessions they attended (number of ANC visits) for their most recent birth. The response options were presented on a continuous scale to ensure a proper understanding of the study outcome. Based on the WHO’s recommendation for eight or more ANC visits [37], which excludes medicals during pregnancy, we categorised the number of ANC visits into 0 = less than eight visits and 1 = eight or more visits. The focus of the study was eight or more ANC visits [38–40].

### Inequality stratifiers

Educational attainment and wealth index were used as inequality stratifiers. Educational attainment entails the highest level of schooling attained by the respondent. The response options were 1 = no education, 2 = incomplete primary, 3 = complete primary, 4 = incomplete secondary; 5 = complete secondary, and 6 = higher. Wealth index on the other hand was computed as a composite index from several household assets [41, 42]. Principal component analysis (PCA) was used to score the various household items for each household member. The PCA was employed due to its ability to reduce data dimensionality while retaining essential information for a study. The resulting scores were categorized into five classes: poorest, poorer, middle, richer, and richest [34,41,42].

### Explanatory variables

Based on an extensive literature search on studies that used the DHS dataset [38,40,43–45], we included fifteen (15) explanatory variables. These variables were the age of the women, current working status, marital status, parity, health insurance coverage, exposure to television, exposure to listening to radio, and exposure to reading newspapers or magazines. Others were internet usage, getting medical help for self: permission to go, getting medical help for self: distance to a health facility, getting medical help for self: getting money for treatment, getting medical help for self: not wanting to go alone, sex of household head, and place of residence. Table 2 presents the various categories of each explanatory variable included in the study.

**Table 2:** Bivariable analysis of ANC visits among women in SSA.

Variables.	n (%)	Eight or more ANC visits % (95% CI) 8.9 [8.5, 9.3]
**Women’s age (years)**		
15–19	8,495 (7.6)	5.7 [5.1, 6.4]
20–24	23,935 (21.3)	7.3 [6.8, 7.8]
25–29	28,285 (25.2)	9.2 [8.7, 9.7]
30–34	22,982 (20.5)	10.3 [9.7, 11.0]
35–39	17,412 (15.5)	10.4 [9.7, 11.1]
40–44	8,242 (7.4)	9.6 [8.6, 10.7]
45–49	2,772 (2.5)	6.6 [5.6, 7.6]
**Educational attainment**		
No education	47,055 (42.0)	5.1 [4.7, 5.4]
Incomplete primary	19,558 (17.4)	5.3 [4.8, 5.7]
Complete primary	9,188 (8.2)	7.8 [6.8, 8.9]
Incomplete secondary	23,039 (20.5)	10.0 [9.3, 10.7]
Complete secondary	8,068 (7.2)	24.8 [23.3, 26.4]
Higher	5,215 (4.7)	29.7 [27.5, 32.0]
**Currently employed**		
No	41,230 (36.8)	6.4 [6.0, 6.8]
Yes	70,893 (63.2)	10.4 [9.9, 10.8]
**Marital status**		
Never in union	9,240 (8.2)	10.5 [9.4, 11.8]
Married	80,629 (71.9)	8.9 [8.5, 9.3]
Cohabiting	15,723 (14.0)	8.7 [7.9, 9.7]
Widowed	1,235 (1.1)	11.6 [9.5, 13.9]
Divorced	2,062 (1.9)	4.4 [3.3, 5.9]
Separated	3,234 (2.9)	7.5 [6.3, 8.8]
**Parity**		
One	24,195 (21.6)	9.9 [9.3, 10.5]
Two-four	54,647 (48.7)	9.9 [9.4, 10.4]
Five or more	33,281 (29.7)	6.6 [6.2, 7.0]
**Covered by health insurance**		
No	99,818 (89.0)	9.0 [8.7, 9.4]
Yes	12,305 (11.0)	7.8 [6.7, 8.9]
**Exposed to watching television**		
No	61,819 (55.1)	6.0 [5.7, 6.4]
Yes	50,304 (44.9)	12.4 [11.8, 13.0]
**Exposed to listening to the radio**		
No	53,314 (47.5)	7.2 [6.8, 7.6]
Yes	58,809 (52.5)	10.5 [10.0, 11.0]
**Exposed to reading newspaper or magazine**		
No	99,058 (88.3)	8.1 [7.7, 8.5]
Yes	13,065 (11.7)	15.2 [14.1, 16.4]
**Used internet**		
No	90,790 (81.0)	7.3 [6.9, 7.6]
Yes	21,333 (19.0)	15.8 [14.8, 17.0]
**Getting medical help for self: Permission to go**		
Not a big problem	89,360 (79.7)	9.4 [9.0, 9.8]
Big problem	22,763 (20.3)	7.1 [6.5, 7.8]
**Getting medical help for self: Distance to a health facility**		
Not a big problem	71,536 (63.8)	10.0 [9.6, 10.5]
Big problem	40,587 (36.2)	6.9 [6.5, 7.5]
**Getting medical help for self: Getting money for treatment**		
Not a big problem	51,462 (45.9)	10.4 [9.9, 10.9]
Big problem	60,661 (54.1)	7.6 [7.2, 8.1]
**Getting medical help for self: Not wanting to go alone**		
Not a big problem	87,915 (78.4)	9.4 [9.0, 9.8]
Big problem	24,208 (21.6)	7.1 [6.5, 7.7]
**Sex of household head**		
Male	90,693 (80.9)	8.7 [8.3, 9.1]
Female	21,430 (19.1)	9.8 [9.2, 10.5]
**Wealth index**		
Poorest	24,159 (21.6)	3.8 [3.4, 4.2]
Poorer	23,792 (21.2)	5.2 [4.8, 5.7]
Middle	22,661 (20.2)	7.5 [7.0, 8.0]
Richer	22,002 (19.6)	11.7 [10.8, 12.7]
Richest	19,509 (17.4)	18.2 [17.1, 19.4]
**Place of residence**		
Urban	44,343 (39.5)	14.3 [13.6, 15.2]
Rural	67,780 (60.5)	5.4 [5.0, 5.7]

### Statistical analyses

Stata software version 17.0 was used to conduct all the analyses. To ensure we applied weighting in our analyses, we divided the women’s weighting variable (v005) by 1000000 to generate a new variable (=v005_pw) in each country’s dataset. Subsequently, the country-level weights were de-normalised using the command: gen v005_pwpool = v005_pw * (total population of women; age 15-49, at the time of the survey/number of women with birth history in the last 5years before the survey [sample]). Later, we appended the dataset for all the countries with all the variables, including the composite weight variable for the analysis. The proportion of eight or more ANC visits across the 15 countries was estimated using percentages. The results of the proportion of women who had eight or more ANC visits were presented using a spatial map. Next, we examined the distribution of eight or more ANC visits across the stratifiers and the explanatory variables using a cross-tabulation table. We presented the results using percentages and confidence intervals (CI). Given that the explanatory variables were numerous, we used the best-variable selection method to select the aggregated list of variables to include in the regression model [46, 47]. To do this, the Stata command “gvselect” was used. The resulting output (group of variables) that had the least Akaike Information Criterion value was chosen as the best-fitted variables for the regression analysis [46, 47]. Before the regression analysis, we checked for evidence of multicollinearity using the variance inflation factor (VIF). The results showed no evidence of multicollinearity with the mean, minimum, and maximum VIFs as 1.38, 1.04, and 1.93, respectively. A weighted multivariable binary logistic regression was performed to examine the factors associated with eight or more ANC visits. The results were presented as adjusted odds ratio (aOR) with their respective 95% CI.

### Inequality analyses

We estimated the degree of wealth and education-related inequalities using concentration curves, concentration indices (CIX), and decomposition analysis. The concentration curve for each of the wealth indices and educational attainment was determined by plotting the cumulative proportion of ANC visits on the y-axis and each of the wealth indices and educational attainment on the x-axis. This was done in increasing order, with the resulting figure showing whether inequality existed or not. The line of equality (45 degrees) was used to assess the extent of inequality in wealth index and educational attainment [48, 49]. Eight or more ANC visits was deemed to be concentrated among the poor or uneducated if the curve was above the line of equality. Similarly, if the curve was below the equality line, eight or more visits were found to be disproportionately concentrated among the rich and educated [48, 49].

Subsequently, the CIX was used to summarise the area between the equality line and the concentration curve. We estimated the CIX using an already developed formula stipulated in the literature [50–52]. The resulting values ranged from − 1 to + 1. With this, a value of zero (0) denoted no evidence of inequality in wealth index or educational attainment. A CIX of 1 indicated that inequalities were concentrated around the richest wealth index or the highly educated individuals whereas a -1 showed an inequality concentrated among the poorest wealth index and the uneducated. We also estimated the CIX and curve per country included in the study.

Later, we examined the individual contribution of the variables included in the study by decomposing the CIX per literature [48]. The results were presented using elasticity, CIX value, absolute contribution, and relative percentage contribution. The elasticity aspect of the results denoted the change in eight or more ANC visits resulting from a unit change in the explanatory variables, with a positive value indicating an increasing trend in ANC visits and a negative value showing a decreasing trend. In addition, a positive CIX value showed that eight or more ANC visits were concentrated in the richer groups or educated individuals whilst a negative value depicted a concentration among the poor and uneducated women. Relative percentage contribution denoted the relative contribution of each model component to the overall wealth and education-related inequality in eight or more ANC visits. As a result, a positive value showed an increase in wealth and education-related inequality, whereas a negative value demonstrated a decrease in inequality.

### Ethical consideration

Ethical clearance was not sought since the study used a secondary dataset, which is available in the public domain. Permission to use the dataset was sought from the Monitoring and Evaluation to Assess and Use Results Demographic and Health Surveys (MEASURE DHS) before the study.

## Results

### Prevalence of eight or more ANC visits among women in SSA

**Fig 1:**
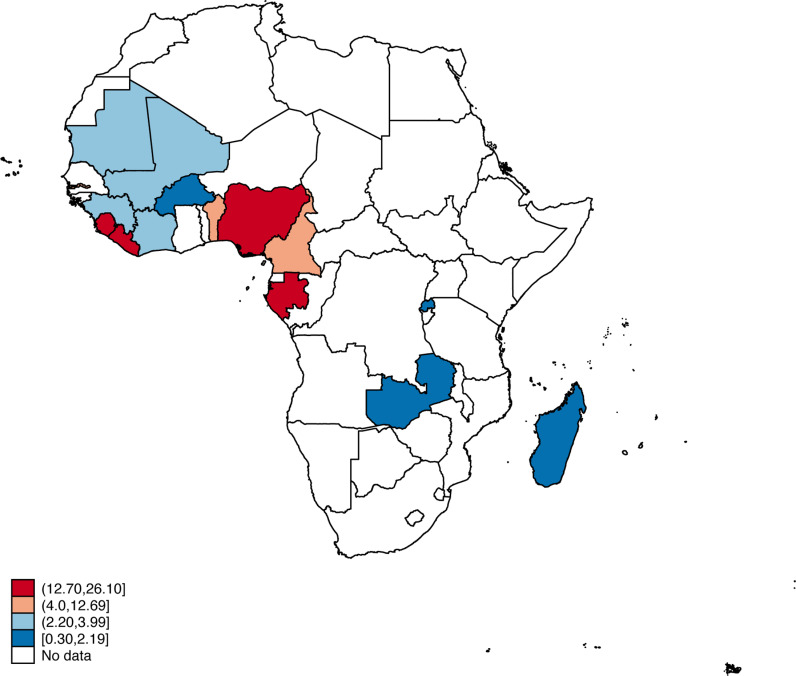
Prevalence of eight or more ANC visits in SSA. Source: Authors, 2024.

Fig 1 shows the spatial distribution of the prevalence of eight or more ANC visits across the fifteen SSA countries. Four countries (Gabon, Liberia, Nigeria, and Sierra Leone) had the highest proportions of eight or more ANC visits, ranging from 12.70% in Gabon to 26.10% in Liberia. Overall, the proportion of eight or more ANC visits among the women in SSA was 8.9% [8.5, 9.3] (Table 2).

### Distribution of eight or more ANC visits across the explanatory variables

Table 2 shows the bivariable results of eight or more ANC visits among women in SSA. The results showed that eight or more ANC visits varies across age groups. The highest prevalence was noted among women aged 35-39 (10.4% [9.7, 11.1]) and the lowest among women aged 15-19 (5.7% [5.1, 6.4]). Women who were identified as having higher education (29.7% [27.5, 32.0]), currently employed (10.4% [9.9, 10.8]), widowed (11.6% [9.5, 13.9]), and not covered by health insurance (9.0% [8.7, 9.4]) had higher prevalence of ANC visits. Differences in eight or more ANC visits were also observed among the various categories of the other variables such as exposure to media, barriers to healthcare access, sex of household head, and wealth index.

### Factors associated with eight or more ANC visits among women in SSA

**Fig 2:**
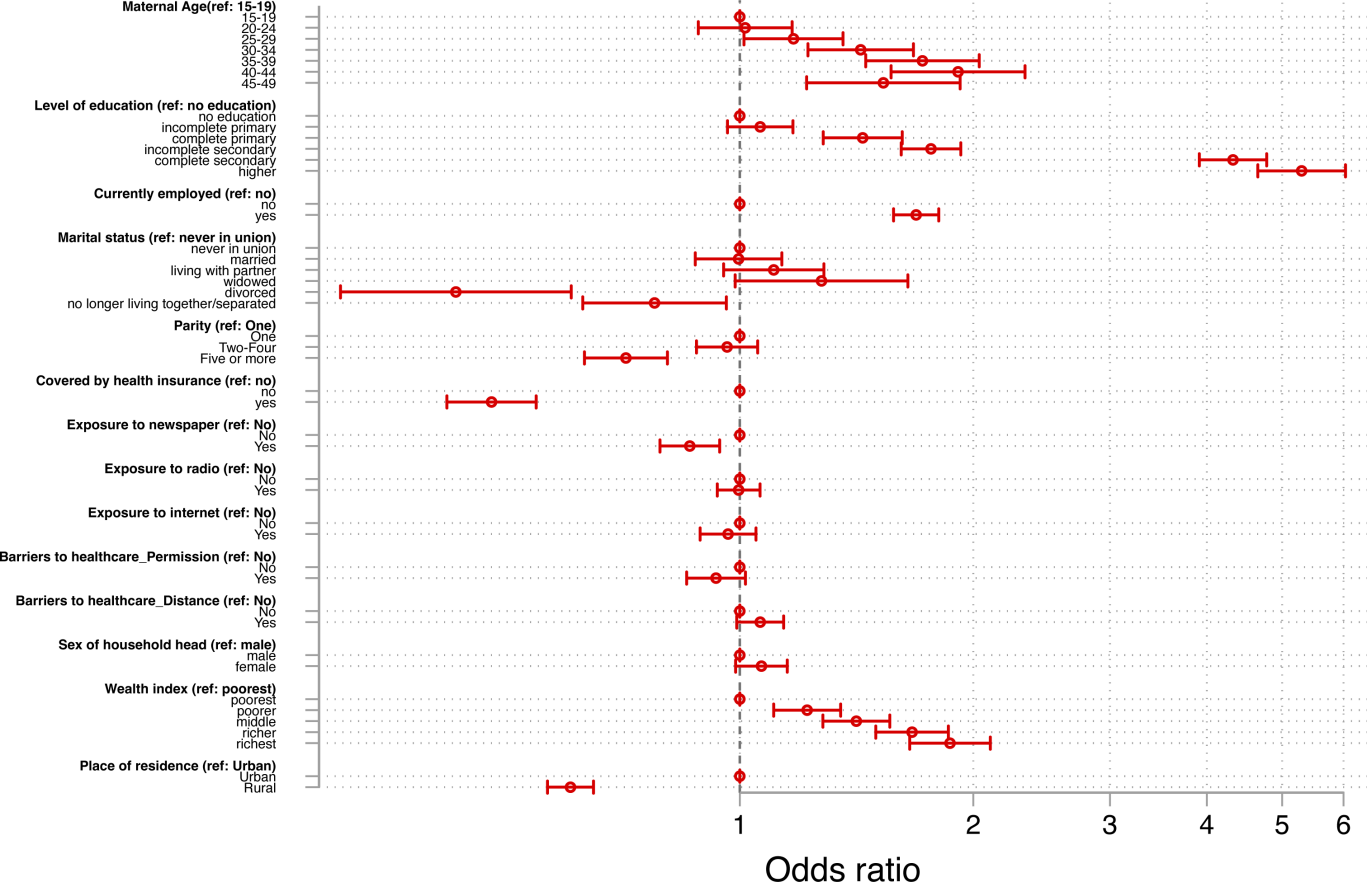
Factors associated with eight or more ANC visits.

Older women were more likely to have eight or ANC visits compared to younger women, with women aged 40-44 years having the highest odds [aOR =  1.91; 95% CI: 1.57, 2.33]. Women who had complete primary education and above showed higher odds for eight or more ANC visits than those without education, with the highest odds among those with higher education [aOR =  5.30; 95% CI: 4.65, 6.03]. Women currently working [aOR =  1.69; 95% CI: 1.58, 1.80] were more likely to have eight or more ANC visits than those who were not working. The odds of eight or more ANC visits increased with increasing wealth indices, with the highest likelihood among women from the richest wealth index households [aOR = 1.87; 95% CI: 1.66, 2.10]. Divorced [aOR = 0.43; 95% CI: 0.31, 0.61] and separated [aOR = 0.78; 95% CI: 0.63, 0.96] women had lower odds for eight or more ANC visits compared to those who were never in a union. Mothers with five or more births [aOR = 0.71; 95% CI: 0.63, 0.81] showed lower odds for eight or more ANC visits relative to those with primiparity. The odds of eight or more ANC visits was lower among women covered by health insurance [aOR =  0.48; 95% CI: 0.42, 0.55] compared to those not covered by health insurance. Lower odds of eight or more ANC visits were observed among women exposed to reading newspapers or magazines [aOR =  0.86; 95% CI: 0.79, 0.94] and rural dwellers [aOR =  0.60; 95% CI: 0.57, 0.65] were less likely to have eight or more ANC visits compared to those who were not exposed to it and urban dwellers, respectively (Figure 2). Details of the regression results are in S2 Table.

### Inequalities in eight or more ANC visits by wealth index

**Fig 3:**
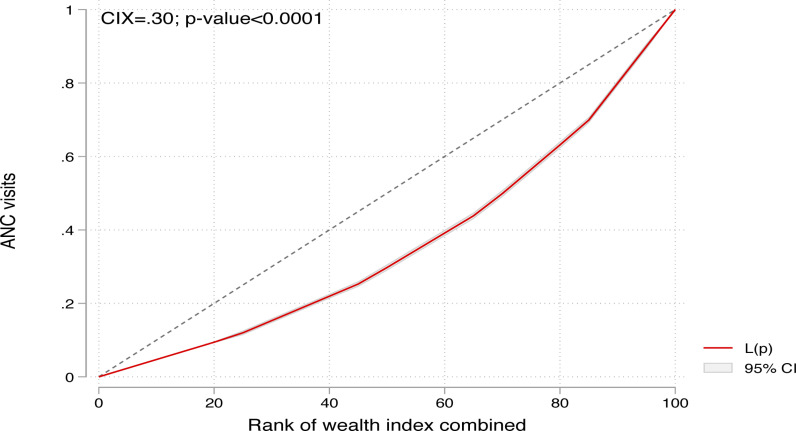
Concentration curves showing inequality in ANC visits by wealth index.

**Fig 4:**
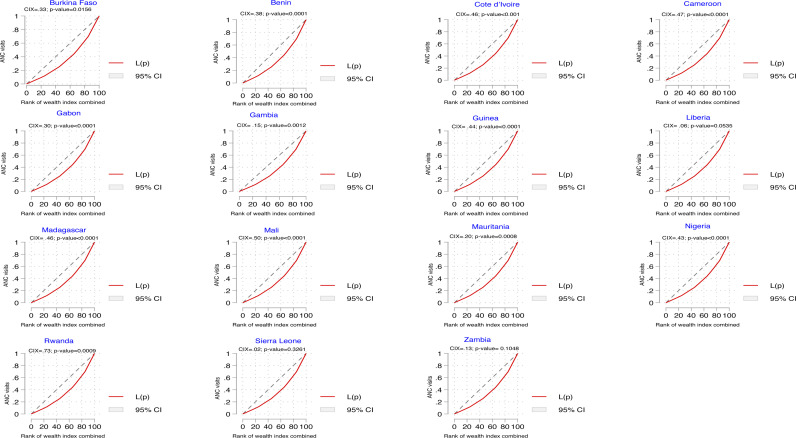
Concentration curves showing inequalities in ANC visits by wealth index in each of the fifteen countries.

The CIX and concentration curve of the wealth-related inequalities in eight or more ANC visits are shown in Fig 3. The positive and statistically significant CIX = 0.30; p-value < 0.0001, and the curve underneath the line in the black diagonal dotted line (line of equality) indicate that eight or more ANC visits are concentrated among women in households with higher wealth index. Consistent results were obtained for all fifteen countries, but these were insignificant in Liberia, Sierra Leone, and Zambia (Fig 4).

### Decomposition analysis of factors contributing to the wealth-related inequalities in ANC visits

Table 3 displays results from the decomposition analysis of factors associated with wealth-related inequalities in eight or more ANC visits. The results are presented in terms of elasticity, CIX, absolute contribution, and percentage contribution. With percentage contribution, educational attainment, wealth index, and place of residence had the highest contribution to the wealth-related inequalities in ANC visits. Together, these variables contributed approximately 32% to the wealth-related inequalities in eight or more ANC visits. Educational attainment accounted for nearly 13% of the wealth-related inequalities in eight or more ANC visits. Women with higher educational levels were responsible for 5.10% of the observed inequality. Positive concentration indices, indicating being advantaged with eight or more ANC visits were found among women with complete primary (CIX = 0.044), incomplete secondary (CIX = 0.229), complete secondary (CIX = 0.468), and higher (CIX = 0.673) level of education. Approximately 12% of the wealth-related inequalities in ANC visits were explained by the wealth index, with the richest wealth index having the highest percentage contribution (8.76%). In terms of the concentration indices, women of the richest (CIX = 0.826), richer (CIX = 0.456), and middle (CIX = 0.057) wealth indices were advantaged in terms of the eight or more ANC visits. Place of residence contributed approximately 8% to the inequalities in eight or more ANC visits. However, women in rural areas were disadvantaged with eight or more ANC visits (CIX = -0.257).

**Table 3:** Wealth-related inequalities in eight or more ANC visits.

Variable	Elasticity	Concentration index	Absolute contribution	Percentage contribution
**Wealth index**				
Poorest	Reference			
Poorer	0.013	−0.357	−0.004	−1.478
Middle	0.021	0.057	0.001	0.392
Richer	0.030	0.456	0.014	4.466
Richest	0.032	0.826	0.027	8.756
**Women’s age (years)**				
15–19	Reference			
20–24	0.001	−0.016	0.000	−0.005
25–29	0.012	0.029	0.000	0.112
30–34	0.022	0.048	0.001	0.345
35–39	0.025	0.018	0.000	0.146
40–44	0.014	−0.047	−0.001	−0.219
45–49	0.003	−0.164	−0.001	−0.168
**Educational attainment**				
No education	Reference			
Incomplete primary	0.003	−0.113	0.000	−0.117
Complete primary	0.009	0.044	0.000	0.129
Incomplete secondary	0.035	0.229	0.008	2.607
Complete secondary	0.031	0.468	0.015	4.810
Higher	0.023	0.673	0.016	5.100
**Currently employed**				
No	Reference			
Yes	0.098	−0.005	−0.001	−0.166
**Marital status**				
Never in union	Reference			
Married	−0.001	−0.005	0.000	0.001
Cohabiting	0.004	−0.028	0.000	−0.039
Widowed	0.001	−0.075	0.000	−0.020
Divorced	−0.005	−0.008	0.000	0.013
Separated	−0.002	−0.032	0.000	0.023
**Parity**				
One	Reference			
Two-Four	−0.005	0.050	0.000	−0.089
Five or more	−0.030	−0.159	0.005	1.555
**Covered by health insurance**				
No	Reference			
Yes	−0.024	0.168	−0.004	−1.327
**Exposed to reading newspaper or magazine**				
No	Reference			
Yes	−0.005	0.402	−0.002	−0.680
**Exposed to listening to radio**				
No	Reference			
Yes	−0.001	0.136	−0.000	−0.023
**Used internet**				
No	Reference			
Yes	−0.002	0.455	−0.000	−0.295
**Getting medical help for self: Permission to go**				
Not a big problem	Reference			
Big problem	−0.004	−0.116	0.000	0.163
**Getting medical help for self: Distance to a health facility**				
Not a big problem	Reference			
Big problem	0.007	−0.206	−0.001	−0.442
**Sex of household head**				
Male	Reference			
Female	0.004	0.033	0.000	0.039
**Place of residence**				
Urban	Reference			
Rural	−0.090	−0.257	0.023	7.636
Residual Concentration index			0.209	

Elasticity: Elasticity measures the responsiveness of one variable to changes in another variable; Concentration Index: The CIX quantifies the degree to which a variable is concentrated among a particular subgroup of the population; Absolute Contribution: Absolute contribution refers to the total impact or effect of a particular variable on an outcome, measured in absolute terms rather than relative or percentage terms; Percentage Contribution: Percentage contribution measures the proportion of a particular variable’s impact relative to the total impact of all factors on an outcome

### Inequalities in eight or more ANC visits by educational attainment

**Fig 5:**
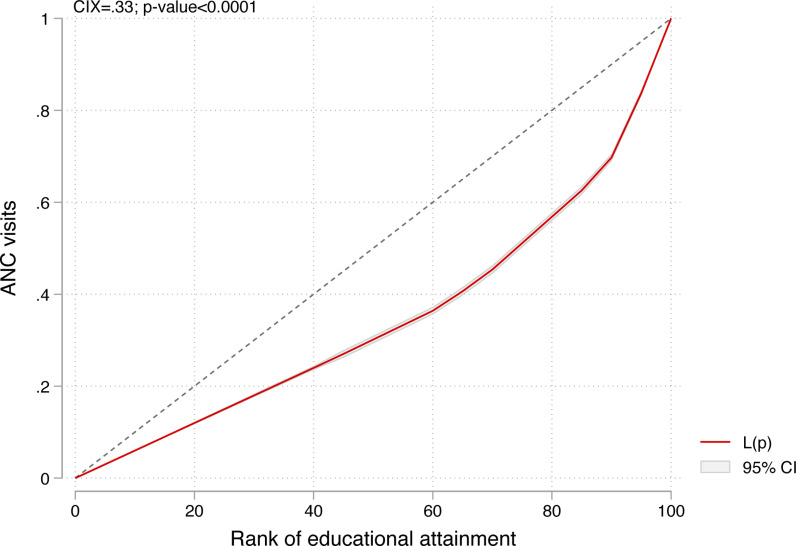
Concentration curve showing inequality in ANC visits by educational attainment.

**Fig 6:**
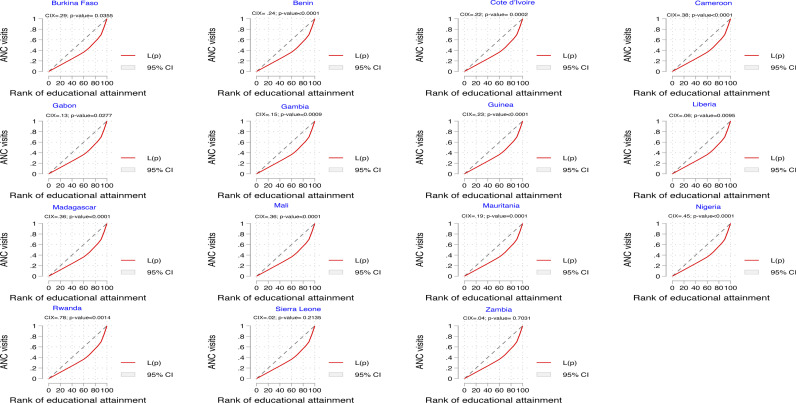
Concentration curves showing inequalities in ANC visits by educational attainment in each of the fifteen countries.

Fig 5 shows the CIX and concentration curve of the educational attainment inequalities in eight or more ANC visits. The positive and statistically significant CIX = 0.33; p-value < 0.0001, and the curve that lies below the line of equality indicate that eight or more ANC visits are concentrated among women in households with higher educational attainment. Consistent results were obtained for all fifteen countries. However, the results were insignificant in Sierra Leone and Zambia (Fig 6).

### Decomposition analysis of factors contributing to the educational attainment-related inequalities in ANC visits

Table 4 indicates results from the decomposition analysis of factors associated with the educational attainment inequalities in eight or more ANC visits. The results are presented in terms of elasticity, CIX, absolute contribution, and percentage contribution. We found that with percentage contribution, educational attainment, parity, wealth index, and place of residence had the highest contribution to the educational attainment inequalities in ANC visits. Collectively, these variables accounted for approximately 33% of inequalities in educational attainment related to eight or more ANC visits. Educational attainment contributed around 21% to the educational attainment disparities in eight or more ANC visits, with women with incomplete secondary education contributing 8.02% to the inequality. Positive concentration indices, indicating being advantaged with eight or more ANC visits, were found among women with incomplete primary (CIX = 0.014), complete primary (CIX = 0.270), incomplete secondary (CIX = 0.558), complete secondary (CIX=0.835), and higher (CIX = 0.953) level of education. Parity contributed nearly 2% to the inequalities in eight or more ANC visits. However, women with grand parity were disadvantaged (CIX = -0.243). Approximately 5% of the educational attainment inequalities in ANC visits were explained by wealth index, with the richest wealth index having the highest percentage contribution (4.28%). In relation to the concentration indices, women of the richest (CIX = 0.433) and richer (CIX = 0.158) wealth indices were advantaged with regards to eight or more ANC visits. Place of residence accounted for 4% disparities in eight or more ANC visits, with women in rural areas disadvantaged (CIX = -0.157).

**Table 4:** Educational attainment inequalities in ANC visits in SSA.

Variable	Elasticity	Concentration index	Absolute contribution	Percentage contribution
**Educational attainment**				
No education	0.003	0.014	0.000	0.013
Incomplete primary	0.009	0.270	0.002	0.736
Complete primary	0.035	0.558	0.019	5.926
Incomplete secondary	0.031	0.835	0.026	8.017
Complete secondary	0.023	0.953	0.022	6.741
Higher				
**Women’s age (years)**	Reference			
15–19	0.001	0.084	0.000	0.026
20–24	0.012	0.056	0.001	0.207
25–29	0.022	−0.005	0.000	−0.032
30–34	0.025	−0.089	−0.002	−0.680
35–39	0.014	−0.156	−0.002	−0.676
40–44	0.003	−0.277	−0.001	−0.266
45–49				
**Currently employed**	Reference			
No	0.098	−0.003	0.000	−0.091
Yes				
**Marital status**	Reference			
Never in union	−0.001	−0.061	0.000	0.015
Married	0.004	0.060	0.000	0.077
Cohabiting	0.001	−0.085	0.000	−0.021
Widowed	−0.005	0.066	0.000	−0.093
Divorced	−0.002	0.171	0.000	−0.114
Separated				
**Parity**	Reference			
Primiparity	−0.005	0.050	0.000	−0.085
Multiparity	−0.030	−0.243	0.007	2.222
Grandparity				
**Covered by health insurance**	Reference			
No	−0.024	0.366	−0.009	−2.697
Yes	0.003	0.014	0.000	0.013
**Exposed to reading newspaper or magazine**			
No	Reference			
Yes	−0.005	0.584	−0.003	−0.920
**Exposed to listening to radio**				
No	Reference			
Yes	−0.001	0.095	−0.000	−0.015
**Used internet**				
No	Reference			
Yes	−0.002	0.474	−0.001	−0.287
**Getting medical help for self: Permission to go**				
Not a big problem	Reference			
Big problem	−0.004	−0.115	0.001	0.150
**Getting medical help for self: Distance to a health facility**				
Not a big problem	Reference			
Big problem	0.007	−0.119	−0.001	−0.238
**Wealth index**				
Poorest	Reference			
Poorer	0.013	−0.171	−0.002	−0.662
Middle	0.021	−0.021	0.000	−0.133
Richer	0.030	0.158	0.005	1.441
Richest	0.032	0.433	0.014	4.284
**Sex of household head**				
Male	Reference			
Female	0.004	0.128	0.000	0.143
**Place of residence**				
Urban	Reference			
Rural	−0.090	−0.157	0.014	4.350
Residual concentration index			0.230	

Elasticity: Elasticity measures the responsiveness of one variable to changes in another variable; Concentration Index: The CIX quantifies the degree to which a variable is concentrated among a particular subgroup of the population; Absolute Contribution: Absolute contribution refers to the total impact or effect of a particular variable on an outcome, measured in absolute terms rather than relative or percentage terms; Percentage Contribution: Percentage contribution measures the proportion of a particular variable’s impact relative to the total impact of all factors on an outcome

## Discussion

Our study assessed the inequalities in eight or more ANC services utilisation among women in SSA, focusing on the influence of wealth and education. This study demonstrates a notable socio-economic disparity in the use of ANC in SSA, with women from more affluent and educated socio-economic backgrounds reporting a considerably higher number of ANC visits than their counterparts with lower socio-economic status. The decomposition analysis identifies critical social factors contributing to inequalities in utilising ANC. These factors include women’s education, wealth, parity, and place of residence.

Our report on ANC service use and higher wealth index aligns with the findings of earlier studies [14–16,18,19,21,24,25]. Affluent women possess the financial resources to cover the expenses related to healthcare services such as transportation, consultation fees, and drugs [53]. They have a higher possibility of being exposed to media and other sources of information that advocate for the utilisation of ANC [53]. A higher socio-economic status is frequently linked to superior education, resulting in increased awareness of the significance of ANC [53]. Affluent women may possess greater autonomy in making healthcare choices, such as accessing ANC services without requiring consent from others [53].

The finding about ANC utilisation and educational achievement corroborates observations of earlier studies [19,54–59]. Women with higher education are more likely to be knowledgeable about the advantages of ANC and the potential dangers of not obtaining it [53]. Education enables women to make well-informed choices and decisions regarding their health and access to essential resources [53]. Women with higher levels of education are more inclined to comprehend medical advice and recognise the significance of adhering to it for the welfare of both them and their unborn children [53,60]. Women with higher levels of education may possess extensive social networks that could actively promote and support the utilisation of antenatal care services [53]. They have a higher probability of being exposed to media and other sources of information that advocate for the utilisation of ANC [53]. Possible strategies to enhance ANC utilisation among women with no education may involve implementing community-based educational initiatives, enhancing the availability of healthcare facilities, and implementing policies to alleviate the financial constraints associated with healthcare [61].

Our study revealed that women who have given birth to several children may face challenges in obtaining ANC services in SSA, particularly when compared to women who have only given birth once. The results of our research validate conclusions drawn in previous studies [19,56,62–65]. Women with grand parity may encounter more significant financial limitations, which might pose challenges in affording the expenses related to ANC visits [66]. Mothers with several children may have limited time to attend ANC appointments due to concerted efforts invested in managing their household. Experienced mothers may perceive reduced susceptibility to problems, potentially leading them to underestimate the importance of ANC [66]. Overwhelmed healthcare systems may struggle to deliver prompt and high-quality care to everyone, especially individuals with more significant needs, such as women with several children [66]. Cultural norms that require experienced mothers to depend on traditional knowledge and practices instead of formal healthcare facilities may also have detrimental effects on child and maternal health outcomes due to a lack of ANC utilisation [67]. To tackle these issues, a comprehensive strategy is needed that encompasses enhancing economic assistance, healthcare infrastructure, and community education to promote the utilisation of ANC services among women with grand parity [66].

The study indicated that women residing in rural areas of SSA are disadvantaged in obtaining ANC services, compared to their urban counterparts. This finding confirms the findings of previous studies [54,68–73]. Rural women often live a considerable distance from healthcare facilities, which constitutes challenges in accessing ANC services; and may face significant barriers due to the expenses associated with transportation and healthcare services [66]. The quality of maternal healthcare in rural areas may be poor, as opposed to what exists in urban areas; and may negatively impact its utilisation [74]. Women in rural areas may exhibit a preference for traditional birth attendants over professional healthcare services, due to the influence of traditional beliefs and practices [66]. In addition, women residing in rural areas are more likely to lack formal education, and this impacts their knowledge and utilisation of healthcare services [26]. They may also have little media exposure, leading to decreased understanding of the significance of ANC [26,75]. Addressing these challenges requires targeted interventions that consider the unique barriers faced by rural women in accessing ANC, to establish more accessible and equitable ANC services for the disadvantaged population [26].

### Policy and practice implications

Socio-economic disparities exist in ANC utilisation in SSA. Women with higher wealth and education use ANC more than those with lower wealth and educational levels. The government in African countries, including SSA should reduce financial barriers by implementing targeted subsidies, voucher programmes, or community-based financing schemes to make ANC more affordable for all women. It is also necessary to increase healthcare infrastructure and invest in women’s education and adult literacy programmes. This could lead to enhanced awareness of the importance of ANC and improve healthcare-seeking behaviour. To address gender disparities, they should empower women economically and within households, especially female heads of households, empower women with more control over healthcare decisions. There is a need to train and equip community health workers to provide essential ANC services, educate women about ANC benefits, and facilitate referrals to higher-level care. Governments should also collaborate with community leaders, traditional birth attendants, and other influential stakeholders to promote positive campaigns about ANC and address cultural barriers. By focusing on these policy and practice implications, stakeholders can work towards reducing wealth and education-based inequalities in ANC utilisation, ensuring that all women in SSA have access to essential healthcare services during pregnancy.

## Strengths and limitations

The main strength of this study is its utilisation of the recent nationally representative datasets of women of reproductive age in fifteen sub-Saharan African countries to investigate the disparities in ANC. Another strength lies in adopting a more meticulous decomposition analysis to identify the disparities in ANC among women in SSA precisely. Nevertheless, the study is constrained by its reliance on cross-sectional data, which impedes establishing causation. Furthermore, since survey participation is voluntary, some women may be omitted due to their lack of motivation to participate, potentially impacting the sample size. Moreso, this study exclusively examined the inequalities using factors available in the DHS dataset, neglecting other potential cultural and religious barriers that could have substantial effects on the utilisation of ANC. These barriers include cultural beliefs and traditions, stigma and privacy concerns, and religious beliefs and practices. The study is also prone to potential recall bias since some of the experiences of women with ANC could have occurred five years before the surveys were administered. This could impact the accuracy of the data, and we suggest directions for future research that might employ medical records or shorter recall periods. Finally, the results from a pooled data analysis of 15 sub-Saharan African countries may hinder understanding the inequalities within country—specific contexts.

## Conclusions

We found that the disparities in ANC services utilisation in SSA are dependent on wealth and education, highlighting the need to implement a comprehensive approach that addresses these inequalities. It is essential to prioritise the critical social factors contributing to disparities in utilising ANC services such as women’s education, parity, place of residence, and economic status. Also, it is equally essential for countries to actively work towards reducing barriers to accessing healthcare services, including ensuring that these services are readily available, affordable, and culturally acceptable. Tackling these complex and interconnected problems has the potential to address disparities in ANC care provision in SSA and decrease maternal illness and death. Countries should also ensure that women achieve a minimum of eight ANC visits, as recently advised by the WHO, focusing on the quality, timing, and substance of service utilisation. By undertaking these actions, SSA will make significant progress towards achieving the Sustainable Development Goal 3 targets 3.1, 3.2, and 3.8, and ensure no woman is left behind.

## Supporting information

S1 Table STROBE Checklist. (PDF)

S2 Table Supplementary results (Factors associated with eight or more ANC visits). (DOCX)

## Abbreviations

aOR: Adjusted Odds Ratio
ANC: Antenatal Care
CI: Confidence Interval
CIX: Concentration Index
DHS: Demographic and Health Survey
MEASURE DHS: Monitoring and Evaluation to Assess and Use Results Demographic and Health Surveys
MMR: Maternal Mortality Ratio
PCA: Principal Component Analysis
SSA: Sub-Saharan Africa
STROBE: Strengthening the Reporting of Observational Studies in Epidemiology
VIF: Variance Inflation Factor
WHO: World Health Organization.
