# Supplementary material for: Utilisation of eight or more antenatal care visits and its associated socio-economic-related inequalities in sub-Saharan Africa: A decomposition analysis
Source: PLoS One. 2025 Mar 25;20(3):e0312412. doi: 10.1371/journal.pone.0312412 (PMC11936203; doi:10.1371/journal.pone.0312412)
Supplement: S2 Table — (DOCX) [file pone.0312412.s002.docx]

**Table S2: Factors associated with eight or more antenatal care visits in sub-Saharan Africa**

| **Variable** | **aOR [95% CI]** |
| --- | --- |
| **Women’s age (years)** |  |
| 15-19 | 1.00 |
| 20-24 | 1.02 [0.88, 1.17] |
| 25-29 | 1.17^*^ [1.01, 1.36] |
| 30-34 | 1.43^***^ [1.22, 1.67] |
| 35-39 | 1.72^***^ [1.45, 2.04] |
| 40-44 | 1.91^***^ [1.57, 2.33] |
| 45-49 | 1.53^***^ [1.22, 1.92] |
| **Educational attainment** |  |
| No education | 1.00 |
| Incomplete primary | 1.06 [0.96, 1.17] |
| Complete primary | 1.44^***^ [1.28, 1.62] |
| Incomplete secondary | 1.76^***^ [1.61, 1.93] |
| Complete secondary | 4.32^***^ [3.91, 4.78] |
| Higher | 5.30^***^ [4.65, 6.03] |
| **Currently employed** |  |
| No | 1.00 |
| Yes | 1.69^***^ [1.58, 1.80] |
| **Marital status** |  |
| Never in union | 1.00 |
| Married | 1.00 [0.88, 1.13] |
| Cohabiting | 1.11 [0.95, 1.28] |
| Widowed | 1.27 [0.99, 1.65] |
| Divorced | 0.43^***^ [0.31, 0.61] |
| Separated | 0.78^*^ [0.63, 0.96] |
| **Parity** |  |
| One | 1.00 |
| Two - Four | 0.96 [0.88, 1.05] |
| Five or more | 0.71^***^ [0.63, 0.81] |
| **Covered by health insurance** |  |
| No | 1.00 |
| Yes | 0.48^***^ [0.42, 0.55] |
| **Exposed to reading newspaper or magazine** |  |
| No | 1.00 |
| Yes | 0.86^**^ [0.79, 0.94] |
| **Exposed to listening to radio** |  |
| No | 1.00 |
| Yes | 1.00 [0.94, 1.06] |
| **Used internet** |  |
| No | 1.00 |
| Yes | 0.97 [0.89, 1.05] |
| **Getting medical help for self: Permission to go** |  |
| Not a big problem | 1.00 |
| Big problem | 0.93 [0.85, 1.02] |
| **Getting medical help for self: Distance to health facility** |  |
| Not a big problem | 1.00 |
| Big problem | 1.06 [0.99, 1.14] |
| **Sex of household head** |  |
| Male | 1.00 |
| Female | 1.07 [0.99, 1.15] |
| **Wealth index** |  |
| Poorest | 1.00 |
| Poorer | 1.22^***^ [1.11, 1.35] |
| Middle | 1.41^***^ [1.28, 1.56] |
| Richer | 1.67^***^ [1.50, 1.86] |
| Richest | 1.87^***^ [1.66, 2.10] |
| **Place of residence** |  |
| Urban | 1.00 |
| Rural | 0.60^***^ [0.57, 0.65] |
| **Total sample** | **112123** |
| **Pseudo R^2^** | **0.109** |

Exponentiated coefficients; 95% confidence intervals in brackets; ^*^ *p* < 0.05, ^**^ *p* < 0.01, ^***^ *p* < 0.001; aOR = Adjusted Odds Ratio; CI = Confidence Interval
